# Editorial: Sexual selection and environmental change: what do we know and what comes next?

**DOI:** 10.1093/cz/zoab021

**Published:** 2021-03-13

**Authors:** Natalie Pilakouta, Murielle Ålund

**Affiliations:** 1 School of Biological Sciences, University of Aberdeen, Aberdeen AB24 3FX, UK; 2 Department of Ecology and Genetics, Animal Ecology, Evolutionary Biology Centre (EBC), Uppsala University, Norbyvägen 18D 75236 Uppsala, Sweden

Anthropogenic environmental change is the most significant threat to biodiversity in the 21st century. Animal populations are experiencing rapid changes in their biotic and abiotic environment, which impose novel selection pressures on organisms and increase the risk of population extinction. There is thus a pressing need to understand what affects the capacity of populations to respond and adapt to environmental change. Because behavioral traits are very labile, they provide a means of rapidly responding to environmental change (Sih et al. 2011; Tuomainen and Candolin 2011).

Mating behavior, in particular, could be especially important given its role in shaping individual reproductive success and population dynamics. Accordingly, in recent years, there has been increasing interest in how sexual selection may influence a population’s ability to cope with environmental change. The 2 main mechanisms of sexual selection are competition for access to mates (intra-sexual selection) and choice of a mating partner (intersexual selection). It has been suggested that stronger mating preferences for “good genes” could lead to higher quality offspring (Martinossi-Allibert et al. 2019). Indeed, a number of recent studies have shown that strong sexual selection can increase population resilience and reduce the risk of extinction (Cally et al. 2019; Godwin et al. 2020; but see Candolin and Heuschele 2008). Sexual selection could therefore potentially improve a population’s ability to cope with environmental change. Yet, changes in environmental conditions may also alter the strength or direction of sexual selection, thereby leading to complex interactions and eco-evolutionary feedback loops between environmental change and sexual selection (Figure 1; Alpedrinha et al. 2019).

**Figure 1:**
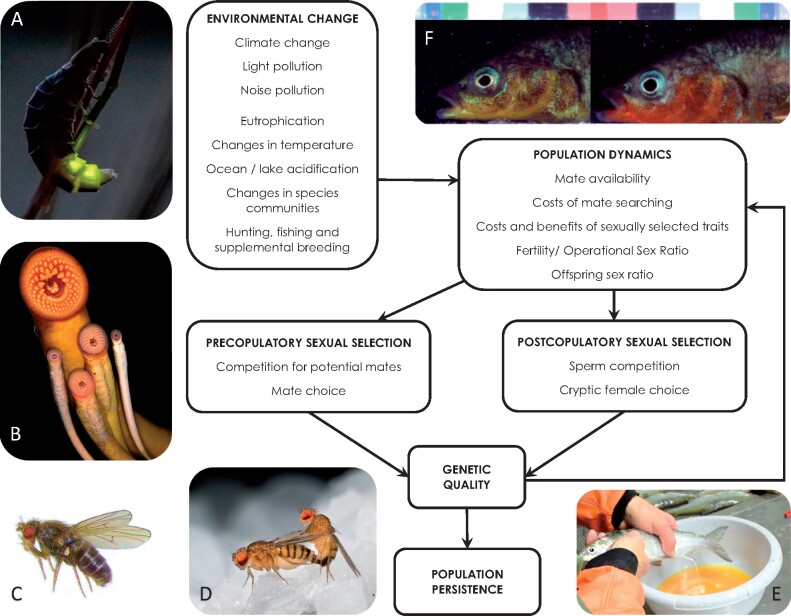
Schematic representation of the links and feedback loop between different sources of environmental change and sexual selection processes, and their consequences for population dynamics and persistence (adapted from Candolin and Heuschele 2008). Photos **A–F** represent the organisms studied in the articles contributed to this special column. Images slightly modified (cropped to size) from the following sources: **A**: Common glow-worm, *L. noctiluca*, by Susanna Kekkonen; **B**: Lamprey species found in the Great Lakes (USA), top: Sea lamprey, *P. marinus*, bottom left to right: American brook lamprey, *Lethenteron appendix*; chestnut lamprey, *I. castaneus*; silver lamprey, *Ichthyomyzon unicuspis*; and northern brook lamprey, *Ichthyomyzon fossor*, by Andrea Miehls; **C**: *Drosophila virilis*, by Nicola White; **D**: Mating fruit flies, *Drosophila melanogaster*, by Francisco Romero Ferrero, licensed with CC BY-SA 4.0, https://creativecommons.org/licenses/by-sa/4.0; **E**: Stripping of milt of a male whitefish, *Coregonus* sp., as part of supplemental breeding procedures in a Swiss hatchery, by Claus Wedekind; **F**: three-spined stickleback, *G. aculeatus*, males with contrasting throat coloration, by Theo C. M. Bakker.

Here, we discuss current empirical and theoretical work on 1) the effects of environmental change on sexual selection and 2) the role of sexual selection in adaptation to environmental change. We then highlight 7 new articles on this topic, published in this special column of *Current Zoology*. We end by identifying some of the major gaps in our knowledge and offer suggestions for future research avenues in this area.

## Effects of Environmental Change on Sexual Selection

Anthropogenic disturbances to natural habitats take many forms and may affect sexual selection in different ways, depending on the organisms’ habitat, physiology, and behavior. Human activity may impact animal communication via changes to the sensory environment, such as light pollution affecting nocturnal species, higher noise levels disrupting mate search and attraction to vocalizations, or eutrophication affecting visual signals in aquatic environments. Theoretical work predicting the effects of environmental change on the strength or direction of sexual selection is still scarce, but a recent model by Martinossi-Allibert et al. (2019) suggests that the strength of sexual selection may often be weakened following rapid environmental change, thereby reducing the putative benefits of sexual selection for adaptation to these new conditions. Despite the dearth of theoretical studies on this topic, empirical studies are rapidly accumulating, and we summarize the main findings of this body of work below. The majority of these have focused on the effects of temperature, reflecting the importance of understanding the profound and wide-ranging impacts of global climate change.

### Effects on mating behavior

In light of higher average temperatures and increased temperature variation due to climate change, numerous studies have focused on viability selection and physiological consequences of heat stress (García-Roa et al. 2020). Nevertheless, changes in ambient temperature can also affect mating behavior, including time searching for mates, courtship behavior, mating rate, and mating duration. For example, fruit flies, *Drosophila melanogaster*, have reduced mating frequencies and mating success under simulated drought conditions (Gefen and Gibbs 2009), while wild populations of sand lizard, *Lacerta agilis*, show increased mating rates and polygyny in warmer years (Olsson et al. 2011). Temperature during mating affects mating duration, sperm transfer, and outcomes of sperm competition in the cigarette beetle *Lasioderma serricorne* (Suzaki et al. 2018). Changes in temperature across a species distribution range may also affect patterns of sexual selection, depending on migration between local populations. For example, worm pipefish, *Nerophis lumbriciformis*, show variation in a range of indicators of sexual selection depending on water temperature (Monteiro et al. 2017).

### Effects on fertility

Recently, a large body of work has focused on the direct impact of increasing temperatures on fertility (reviewed in Walsh et al. 2019), often measured in terms of sperm production. For example, experimental heat waves increase the proportion of abnormally shaped sperm in zebra finches *Taeniopygia guttata*, potentially constraining the birds to narrower windows of time suitable for breeding, which would likely increase competition for mates (Hurley et al. 2018). Several studies in fishes and insects also show temperature-induced differences in sperm shape, velocity, and concentration that may affect the outcome of sperm competition (Breckels and Neff 2013; Vasudeva et al. 2014) or the link between pre- and post-copulatory sexual selection (Iglesias-Carrasco et al. 2020). In the yellow dung fly, *Scathophaga stercoraria*, developmental temperature affects female reproductive morphology, specifically the number of sperm storage organs, with consequences for the outcome of sperm competition and male relative fertilization success (Berger et al. 2011).

Furthermore, there is evidence for sex-specific effects of temperature on fertility with potential implications for operational sex ratios. In *D. melanogaster*, increased developmental temperatures negatively affect male fertility, but females are much less sensitive to this treatment (Zwoinska et al. 2020), suggesting temperature changes may modify operational sex ratio in this species. In mice, heat stress can cause a decrease in sperm viability with a differential effect on Y- and X-bearing sperm, potentially affecting offspring sex ratio (Pérez-Crespo et al. 2008).

### Effects on sexual signals and benefits of mate choice

Changes in environmental conditions may directly or indirectly affect the expression of secondary sexual characters or the fitness benefits of specific mate preferences. For example, increased turbidity following algal blooms causes three-spined stickleback, *Gasterosteus aculeatus*, males to invest more time and energy courting females but with no corresponding increase in successful female attraction (Candolin et al. 2007). In the sand goby *Pomatoschistus minutus*, increased turbidity alters females’ perception of intra-sexual competition, resulting in reduced male reproductive success (Järvenpää et al. 2019). In insects, changes in temperature are known to affect odor profiles, which can influence female mate choice, as observed in the red mason bee *Osmia bicornis* (Conrad et al. 2017). Temperature could also indirectly affect sexual selection through a reduction in condition, influencing the expression of secondary sexual traits (García-Roa et al. 2020). Other indirect effects of environmental change on sexual selection include changes in food availability or predator pressure that may result in less time or energy invested in courtship displays or mate searching and selection (Candolin and Wong 2015).

Moreover, changes in environmental conditions can alter the honesty of a secondary sexual signal, which may affect what proportion of males get an opportunity to reproduce, and which genes are passed onto the next generation (Candolin and Wong 2015). In collared flycatchers *Ficedula albicollis*, the consequences of mate choice vary with climatic conditions during or shortly after the breeding season; females breeding with highly ornamented males experience high fitness in dry years but low fitness in wet years (Robinson et al. 2012). In extreme cases, environmentally induced disruption of sexual selection may directly threaten biodiversity. This is the case of Lake Victoria cichlids, for which species boundaries are usually maintained through assortative mating, but mate choice has been impaired by human-induced eutrophication, resulting in a lower number of species in turbid areas (Seehausen et al. 1997). Lastly, a sudden change in environmental conditions is expected to strongly reduce sexually antagonistic selection, as both sexes will be far away from their optima and selection will thus initially operate in the same direction (Connallon and Hall 2018).

## Effect of Sexual Selection on Adaptation to Environmental Change

Although the effects of environmental change on sexual selection processes have been studied extensively and are now well-established, it is less clear how such changes in sexual selection may in turn influence the capacity of populations to adapt to environmental change. So far, theoretical models and empirical work have provided contradicting predictions and mixed evidence for whether changes in intra-sexual and intersexual selection will moderate or exacerbate the effects of human-induced environmental change.

### Theoretical models

Using an individual-based genetic model, Lorch et al. (2003) investigated the effect of covariance between male condition and display on the rate of adaptation. They show that natural and sexual selection can have synergistic effects that increase population fitness and accelerate the rate of adaptation. They also argue that this feedback between natural and sexual selection could be a particularly potent force under environmental change (Lorch et al. 2003). On the other hand, Fox et al. (2019) argue that an improvement in mean male condition under environmental change may not necessarily have a positive effect on population fitness. Instead, even when environmentally induced changes in the expression of sexually selected male traits increase male fitness, it is possible for female reproductive output to be negatively affected, thereby increasing the risk of population extinction. Such a scenario could arise if, for example, a change in environmental conditions leads to stronger selection for coercive male traits that reduce female fecundity or longevity (Fox et al. 2019). Another recent study used an individual-based model simulating a population evolving under variable environments with different degrees of sexual selection and condition dependence, allowing feedback between demographic and evolutionary processes (Martínez-Ruiz and Knell 2017). The authors show that sexual selection can either increase or decrease average individual fitness in a population depending on the carrying capacity of the environment, the fecundity of individuals in the population, the nature of environmental variation, and the extent of condition dependence of any sexual displays (Martínez-Ruiz and Knell 2017).

### Empirical evidence

A review of empirical studies by Candolin and Heuschele (2008) suggested that sexual selection has no effect, or even a negative effect, on the rate of adaptation to environmental change. This was based on the results of studies using fruit flies *D. melanogaster* and yellow dung flies *S. stercoraria*, which found that the removal of sexual selection improves reproductive rate, possibly due to a reversal of antagonistic coevolution between sexes (Holland and Rice 1999; Martin et al. 2004). Additional studies on bulb mites *Rhizoglyphus robini* and fruit flies (*D. melanogaster* and *D. serrata*) found no evidence that sexual selection influences population fitness during exposure to a novel environment (Holland 2002; Radwan et al. 2004; Rundle et al. 2006; Tilszer et al. 2006). As a result, this review concluded that sexual selection is unlikely to play a substantial role in accelerating adaptation and preventing population extinction (Candolin and Heuschele 2008).

However, since then, a number of studies have shown that sexual selection can indeed be beneficial in novel or rapidly changing environments. For example, sexual selection facilitates adaptation and reduces the rate of extinction in seed beetles *Callosobruchus maculatus* reared on a novel food resource, in flour beetles *Tribolium castaneum* exposed to a pesticide, and in bulb mites *R. robini* exposed to thermal stress (Fricke and Arnqvist 2007; Plesnar-Bielak et al. 2012; Jacomb et al. 2016). Similarly, Parrett and Knell (2018) compared the fitness of Indian meal moth *Plodia interpunctella* populations experiencing either strong or weak sexual selection under exposure to elevated temperatures. They found that stronger sexual selection is associated with increased fecundity and offspring survival, suggesting that sexual selection may have driven adaptive evolution by favoring beneficial alleles (Parrett and Knell 2018). It is worth noting that all of these studies were conducted on insects, which may be, at least partially, due to the practical limitations of running multi-generational laboratory experiments on organisms with long generation times (Miller and Svensson 2014).

## Contributions to This Issue

This special column consists of studies investigating the effects of environmental change on pre-copulatory sexual selection (Buchinger et al. 2021; Elgert et al. 2021; Hiermes et al. 2021a, 2021b), post-copulatory sexual selection (Perroud et al. 2021), or a combination of the 2 (Walsh et al. 2021). In addition, one study (Gómez-Llano et al*.* 2021) addresses the potential roles of pre- and post-copulatory sexual selection for adaptation to temperature changes. Our contributing authors have used a wide range of methods, from measures of trait variability in the wild to laboratory-based behavioral experiments and experimental evolution, to assess the impact of sensory communication disruption (visual and olfactory), physiological stress (temperature), and human-induced changes to population structure (fishing and supplemental breeding) on the strength and outcome of sexual selection.

More specifically, 2 of the studies in this issue focus on temperature and its relationship with different aspects of sexual selection. Walsh et al. (2021) exposed cosmopolitan fruit flies *Drosophila virilis* to sublethal heat shock as pupae and examined the consequences for both male and female fertility over their lifespan. Differences in sexual maturation time and number of offspring produced by heat-stressed males and females resulted in temporary changes in operative sex ratio; this “cryptic sterility” could have important short- and long-term consequences for natural populations. Gómez-Llano et al. (2021) used experimental evolution in *D. melanogaster* to assess how different mating regimes (no mate choice, only pre-copulatory sexual selection, or a combination of pre- and post-copulatory sexual selection) affect adaptation to stable, gradual, and sudden changes in temperature. Their fitness assays suggest a beneficial effect of sexual selection for adaptation to some (but not all) scenarios of climate change, after just a few generations of evolution.

Three other studies in this issue ask how changes in the sensory environment affect sexual signals and mate preferences. By experimentally simulating anthropogenic light pollution in the field, Elgert et al. (2021) show that bright artificial lighting negatively affects mate attraction success in the European common glow-worm, *Lampyris noctiluca*. However, they suggest that sexual selection for the evolution of brighter signals could potentially mitigate the impact of light pollution in this species. Hiermes et al. (2021a, 2021b) focused on female preferences in relation to a little-studied trait (ultraviolet signals) in the three-spined stickleback. Behavioral trials on populations from 2 contrasting light environments tested in 2 visibility conditions show that water conditions affect both the expression of ultraviolet-based traits and female preference for these traits (Hiermes et al. 2021a). The authors then used a common garden design to test shoaling preferences and mating preferences in lab-reared F1 fish originating from these 2 light environments. This follow-up experiment showed that social and mating preferences persist in fish reared under the same conditions, suggesting the presence of parental effects or heritability of these preference patterns (Hiermes et al. 2021b).

Finally, 2 studies investigated the effect of changes in aquatic ecosystems on sexual selection (Buchinger et al. 2021; Perroud et al. 2021). Buchinger et al. (2021) investigated whether heterospecific signals from recently arrived invasive species can interfere with the mating signals of native species. They explored this scenario in the Laurentian Great Lakes, where sea lampreys *Petromyzon marinus* pose a threat to 4 native lamprey species. The authors tested for potential reproductive interference via sex pheromones by exposing native chestnut lamprey *Ichthyomyzon castaneus* to the odors of conspecifics, as well as heterospecific invasive sea lampreys. Their results suggest detrimental consequences of this invasive species through “pheromone pollution,” which could present a serious threat to the successful reproduction of native lamprey species. Lastly, Perroud et al. (2021) asked how common practices of supplemental breeding, where gametes are randomly mixed to produce offspring, differ from natural processes of mate choice and sperm competition in whitefish (*Coregonus* sp.). They discuss how removing a crucial aspect of sexual selection may bias which males get to reproduce, with potential consequences for the genetic make-up of the population.

## Open Questions and Future Challenges

Given the unprecedented rate of human-induced environmental change, studies on the interaction between sexual selection and environmental change have been rapidly accumulating over the past 2 decades. Nevertheless, several fundamental questions remain unanswered, and we believe there are many exciting research avenues still to be explored. In this section, we identify some of these major knowledge gaps and discuss potential challenges for future research.

### Effects of environmental change on different components of sexual selection

Changes in environmental conditions could influence the intensity of competition for access to mates (e.g., through changes in the operational sex ratio), the strength or direction of mating preferences (e.g., through changes in the costs and benefits of mate choice), the outcome of sperm competition (e.g., through effects on sperm number or quality), as well as cryptic female choice (Figure 1). However, we are not aware of any studies that have attempted to partition environmentally induced variation in sexual selection arising from all of these mechanisms (Figure 1). We believe it would be worthwhile for future work on this topic to use integrative approaches that can examine the separate and combined effects of these mechanisms on sexual selection processes under environmental change.

### Effects of multiple environmental stresses on sexual selection

Natural populations are likely to be simultaneously exposed to multiple types of human-induced environmental changes in coming decades (Tuomainen and Candolin 2011). Yet, most studies focus on the effects of just one factor (e.g., temperature, artificial light, noise, or eutrophication) on sexual selection. We strongly encourage future research on this topic to investigate how interactions between different environmental stresses may influence sexual selection and population fitness. For example, future work should determine whether the effects of multiple stresses are additive, compensatory, or multiplicative. While we acknowledge that such studies will require more complex experimental designs and may thus present logistical challenges, we believe they could offer particularly novel and valuable insights by more closely simulating a real-world scenario, where natural populations are often experiencing 2 or more environmental stresses simultaneously.

### Eco-evolutionary feedback loops between sexual selection and environmental change

Our review of the literature suggests that the majority of studies on this topic examine the effects of environmental change on sexual selection, with much fewer studies investigating how sexual selection may facilitate or hinder adaptation to environmental change. In addition, we still lack theoretical and empirical work that explicitly considers the potential eco-evolutionary feedback loop between sexual selection and environmental change, rather than focusing on just one component of this interaction (Figure 1). Empirical studies aiming to address this issue will require multi-generational laboratory experiments or long-term field studies of natural populations responding and adapting to environmental changes.

### Linking individual-level molecular changes to population-level consequences

As reviewed above, an accumulating number of studies have shown that environmental changes can influence mating behavior and thus sexual selection processes. Yet, we lack studies investigating the physiological and molecular mechanisms that underlie these changes. One fruitful avenue for future research would be to examine responses in endocrine pathways and brain gene expression to better understand the proximate basis of environmentally induced changes in mating behavior. Such integrative approaches would allow us to link individual-level molecular changes in behavior to population-level fitness consequences.

### Taxonomic bias

A recent review on the effects of temperature changes on sexual selection noted that studies on this topic are biased toward insects (García-Roa et al. 2020). Part of the reason for this taxonomic bias may be that ectotherms, including insects, are particularly vulnerable to climate change. This is because changes in ambient temperature directly influence their body temperature, and in turn their metabolic rate and capacity to perform behavior (Brandt et al. 2020), as well as posing challenges to their fertility (Walsh et al. 2019). In light of this, we emphasize the need to also better understand the effects of temperature changes on sexual selection in other ectothermic animals, such as fishes, reptiles, and amphibians.

We also highlighted above that so far, experimental studies examining the role of sexual selection in adaptation to environmental change were all conducted using insect study systems, such as fruit flies, bulb mites, flour beetles, and seed beetles (Holland 2002; Radwan et al. 2004; Rundle et al. 2006; Tilszer et al. 2006; Fricke and Arnqvist 2007; Plesnar-Bielak et al. 2012; Jacomb et al. 2016). We recognize that the short generation times associated with these study systems make them well-suited for such experimental evolution studies. Nevertheless, we encourage researchers to also take advantage of other non-insect model systems with relatively short generation times (e.g., fishes: zebra fish; birds: zebra finches) to get a wider taxonomic representation of the links between environmental change and sexual selection.

## Concluding Remarks

Human activity can cause changes in many aspects of an organism’s abiotic and biotic environment, for example through changes in temperature, light, visibility, and ambient noise, or community-level changes affecting competition for resources or predation pressure. Studies across diverse taxa have shown a multitude of consequences of these environmental changes on mating behavior, the expression of secondary sexual traits, and fertility, with consequences for the pattern, reliability, and efficacy of sexual selection. Sexual selection has in turn been predicted to either facilitate or hinder adaptation to changing environments.

Our editorial and the studies contributed to this special column highlight that there are still many unexplored aspects of the relationship between sexual selection and environmental change. We strongly encourage future research to investigate multiple sources of environmental disturbances and/or multiple stages of sexual selection, while widening the taxonomic range of study organisms being used. Such work would greatly advance our understanding of the consequences of environmental change on sexual selection, as well as the potential for sexual selection to influence adaptation to these new conditions in a rapidly changing world.
